# Prevalence and Clinical Correlates of Comorbid Anxiety and Panic Disorders in Patients with Parkinson’s Disease

**DOI:** 10.3390/jcm10112302

**Published:** 2021-05-25

**Authors:** Camilla Elefante, Giulio Emilio Brancati, Silvia Bacciardi, Sonia Mazzucchi, Eleonora Del Prete, Giovanni Palermo, Daniela Frosini, Ubaldo Bonuccelli, Roberto Ceravolo, Lorenzo Lattanzi, Icro Maremmani, Giulio Perugi

**Affiliations:** 1 2nd Psychiatric Unit, Department of Clinical and Experimental Medicine, Santa Chiara University Hospital, University of Pisa, 56100 Pisa, Italy; camifele@gmail.com (C.E.); giuliobrancati@gmail.com (G.E.B.); s.baciard@gmail.com (S.B.); llattanzi@blu.it (L.L.); giulio.perugi@med.unipi.it (G.P.); 2 Neurological Unit, Department of Clinical and Experimental Medicine, Santa Chiara University Hospital, University of Pisa, 56100 Pisa, Italy; mazzucchi.s@gmail.com (S.M.); e.delprete86@hotmail.it (E.D.P.); palermo.giovanni85@gmail.com (G.P.); danielafrosini80@gmail.com (D.F.); u.bonuccelli@med.unipi.it (U.B.); r.ceravolo@med.unipi.it (R.C.)

**Keywords:** Parkinson disease, movement disorder, neurodegenerative diseases, anxiety disorders, panic disorder, mood disorders

## Abstract

Mood and anxiety disorders are the most common neuropsychiatric syndromes associated with Parkinson’s disease (PD). The aim of our study was to estimate the prevalence of lifetime and current anxiety disorders in patients with Parkinson’s Disease (PD), to explore possible distinctive neurological and psychiatric features associated with such comorbidity. One hundred patients were consecutively recruited at the Movement Disorders Section of the Neurological Outpatient Clinic of the University of Pisa. According to the MINI-Plus 5.0.0, 41 subjects were diagnosed with lifetime anxiety disorder (22 with panic disorder) and 26 were diagnosed with current anxiety disorders. Patients with anxiety disorders were more frequently characterized by psychiatric symptoms preceding PD, lifetime major depression and antidepressant treatments. They showed more anxious temperamental traits and scored higher at Parkinson Anxiety Scale (PAS) and persistent anxiety subscale. Current anxiety disorders were associated with more severe psychopathology, depressive symptomatology, and avoidant behavior. Among anxiety subtypes, patients with lifetime panic disorder showed higher rates of psychiatric symptoms before PD, lifetime unipolar depression, current psychiatric treatment, and a more severe psychopathology. Given the overall high impact of anxiety on patients’ quality of life, clinicians should not underestimate the extent of different anxiety dimensions in PD.

## 1. Introduction

Parkinson’s disease (PD) is the second most common age-related neurodegenerative disorder after Alzheimer’s disease. The number of people affected by PD worldwide was estimated around four millions in 2005 and, given the population aging, this number is expected to more than double by the year 2030 [1]. The clinical diagnosis of PD is based on the presence of cardinal motor signs (bradykinesia, rigidity, rest tremor) [2], however PD is now better conceptualized as a neuropsychiatric disorder rather than as a movement disorder. In fact, a wide range of psychiatric symptoms, cognitive dysfunctions and other non-motor manifestations are associated with motor features [3,4].

Neuropsychiatric symptoms (NPS) in PD are currently considered as a source of disability comparable to motor symptoms. Higher frequency and severity of NPS was found to be significantly associated with poorer outcome and higher caregiver burden [3]. Sometimes NPS precede the diagnosis of PD, but more frequently emerge with advancing age and disease severity [5]. Mood and anxiety disorders are the most common neuropsychiatric syndromes associated with PD, with prevalence respectively estimated up to 50% [6] and 40% [7,8,9].

Although major depression is the most studied psychiatric disorder in PD [6], anxiety disorders also deserve attention. Anxious symptoms are even more prevalent than depression in PD and are significantly associated with quality of life [10]. Only in recent years prevalence and features of anxiety disorders in PD have been investigated. Anxiety disorders resulted much more frequent in PD population compared to both adults and elderly controls [11,12,13] and also to patients affected by other chronic somatic diseases [14,15,16,17]. However, a large proportion of anxious PD patients remain unrecognized [18], because anxiety in PD exhibits a complex and heterogenous symptomatology that not always meets diagnostic criteria for a typical anxiety disorder [19]. PD-specific anxiety symptoms include distress, worry, fear, agitation, embarrassment, and social withdrawal associated with motor disability and complications arising from PD medication [19]. Moreover, many anxious symptoms overlap with motor, autonomic and cognitive features of PD, further complicating the diagnosis and treatment of anxiety disorders [9,20].

The aim of our study was to estimate the prevalence of lifetime and current anxiety disorders in a relatively large sample of adult patients affected by PD admitted at the Movement Disorders Section of the Neurological Outpatient Clinic of the University of Pisa. We expected anxiety disorders to be highly represented in our sample. Moreover, we speculated that anxiety disorder patients had earlier onset of PD, higher severity of other non-motor symptoms, higher anxiety and depression ratings, higher rates of comorbidities with mood disorders and more psychiatric treatments compared to patients without anxiety.

## 2. Materials and Methods

One hundred adult patients with PD were consecutively recruited at the Movement Disorders Section of the Neurological Outpatient Clinic of the University of Pisa within the period between October 2017 and March 2018. Neurological evaluations have been carried out by a movement disorder specialist. All participants received the diagnosis of probable PD. If the onset of the disease occurred after 2015, the Movement Disorder Society criteria were applied [2]. If instead the onset of the disease was before 2015 the diagnosis was made with the UK Parkinson’s Disease Brain Bank criteria [21] and then confirmed according to the new criteria. Approval from the local ethical committee was obtained and all data were collected in accordance with the Helsinki Declaration.

Information about the neurological disorder, including PD age of onset, side of onset, PD subtype, duration of illness, presence of motor fluctuations, involuntary movements and gross neuroimaging alterations (i.e., hyperintensities/lacunes, atrophy, enlargement of the ventricles/hydrocephalus), was retrieved from clinical records collected during routine visits by expert neurologists. The third section from the Unified Parkinson’s Disease Rating Scale (UPDRS) [22], a 42-item scale used in the clinical evaluation of PD, was routinely completed by the examining neurologist for the evaluation of motor severity. Cognitive function was explored through Mini-Mental State Examination (MMSE). Patients were all evaluated in the therapeutic ON state. Socio-demographic data (age and gender, marital status, education and profession) and psychiatric history information were collected by psychiatrists with at least 2 years of experience (CE, SB) in the context of individual interviews with each patient and caregiver. The diagnosis of lifetime anxiety disorders and lifetime depressive disorder were assessed through the Mini International Neuropsychiatric Interview (MINI)-Plus version 5.0.0 [23], that adheres to the DSM-IV-TR criteria [24]. According to MINI-Plus, both lifetime and current diagnoses of panic disorder and agoraphobia were assessed, while the diagnoses of generalized anxiety disorder (GAD), social anxiety disorder and specific phobias were only made cross-sectionally at the time of the interview. Thus, both patients with current anxiety disorders and patients who have ever satisfied criteria for panic disorder and/or agoraphobia over the course of life were included among patients with lifetime anxiety disorders. Other assessment instruments and further details on recruitment were previously described by Bacciardi et al. [25].

A series of validated questionnaires was administered, namely, Parkinson Anxiety Scale (PAS), observer-rated version [26,27], Beck Depression Inventory-II (BDI-II) [28], Semi-structured Interview for Mood Disorders (SIMD-R) [29], Temperament Evaluation of Memphis, Pisa, Paris and San Diego-Münster (TEMPS-M) [30], Brief Psychiatric Rating Scale 4.0 (BPRS 4.0) [31], and Non-Motor Symptoms Scale for Parkinson’s Disease (NMSS) [5]. The PAS, observer-rated version, was used to investigate anxiety symptoms occurring in the four weeks prior to the interview. It consists of 12 items subdivided into three domains: persistent anxiety, episodic anxiety and avoidant behavior. Questions and answering options for every item are read out to the patient. Each question is coded on a 5-point Likert scale (0 meaning “no or never” and 4 meaning “severe or almost always”) [27]. The presence of depressive symptoms in the weeks prior to the interview, was also evaluated with the BDI-II, a self-report questionnaire consisting of 21 items scored 0 to 3 [28]. Bipolar spectrum disorders were diagnosed with the SIMD-R, which has been designed for and widely used in clinical research with bipolar patients [32,33]. Lifetime impulse control disorders (ICDs) such as gambling disorder, compulsive sexual behavior, compulsive buying, were clinically assessed according to DSM-IV-TR [24], where the latter two disorders are subsumed under the ICDs not otherwise specified diagnostic category. In addition, according to Weintraub & Claassen proposal [34], binge-eating disorder was also considered among ICDs and was diagnosed according to DSM-IV-TR criteria [24]. The temperamental characteristics were assessed by means of the TEMPS-M, a self-evaluation form of 35 items coded on a 5-point Likert scale (from absent to very much) and including five subscales, one for each affective temperamental disposition, namely depressive, cyclothymic, hyperthymic, irritable and anxious [30]. Finally, psychopathological evaluation was completed using BPRS, which provides a structured mental state examination form covering, in 24 items rated 1 to 7, relatively independent syndromic dimensions mainly related to affective, anxiety and psychotic disorders [31]. The NMSS, instead, investigates non-motor symptoms in PD in general and consists of 30 items divided into 9 domains: cardiovascular, sleep/fatigue, mood/apathy, perceptual disorders/hallucinations, attention/memory, gastrointestinal symptoms, urinary symptoms, sexual function, miscellaneous. Questions refer to the month before the interview and the score for each item is based on a combination of severity (from 0 to 3) and frequency (from 1 to 4). The total score ranges from 0 to 21 [5].

Statistical analyses were performed using R Statistical Software (Foundation for Statistical Computing, Vienna, Austria). In order to explore the distinctive neurological and psychiatric features associated with anxiety disorders, the sample was further subdivided in two different ways in three groups. First, patients without any diagnosis of anxiety disorders were compared with patients with current anxiety disorders and patients with previous remitted anxiety disorder. Secondly, the differential role of episodic (vs. persistent or situational) anxiety were investigated, comparing patients without any diagnosis of anxiety disorders with patients with lifetime panic disorder and patients with other lifetime anxiety disorders. Finally, we tested the associations between anxiety symptoms severity measured by PAS and motor signs severity measured by UPDRS part III, general cognition estimated by MMSE, non-motor symptoms of PD described in NMSS domains, and general psychopathology, depressive symptoms and affective temperamental dispositions, respectively assessed by BPRS, BDI-II and TEMPS-M.

Clinical neurological and psychiatric differences among subgroups were investigated by means of Pearson’s chi-square test (or Fisher’s exact test when appropriate) for categorical variables and Kruskal-Wallis test for continuous ones, after excluding normality using Shapiro-Wilk test, and for ordinal variables (i.e., rating scales). Pairwise Fisher’s exact test and Dunn’s test of multiple comparisons were respectively used for post-hoc comparisons, using Benjamini-Hochberg procedure as false discovery rate (FDR) correction method. Spearman’s rho was used for correlational analyses. *p*-values < 0.05 were considered indicative of statistical significance.

## 3. Results

### 3.1. Sample Characteristics

The sample consisted of 100 patients of which 39 (39%) were females. The average age was 67.2 ± 10.6, ranging from 37 to 90 years. According to MINI-Plus and SIMD-R, 71 (71%) patients had at least one comorbid psychiatric disorder. Similarly, 61 patients were taking a psychopharmacological therapy in addition to antiparkinsonian therapy. According to MINI-Plus, 42 patients had a history of a depressive episode, 26 of which met criteria for major depressive disorder. As previously reported, bipolar spectrum disorders (i.e., including drug-induced hypomania and unipolar hypomania) were diagnosed in 32 patients according to SIMD-R [25]. Overall, 26 (26%) patients were currently diagnosed with at least one anxiety disorder according to MINI-Plus, with GAD being the most frequent current anxiety disorder (15 of 100, 15%). The prevalence of lifetime anxiety disorders settled at 41% (*n* = 41), with lifetime panic disorder being the most common condition (22 of 100, 22%) (Table 1).

### 3.2. Comparisons among Patients without Anxiety Disorders, with Past Anxiety Disorders and with Current Anxiety Disorders According to MINI-Plus

Patients without any lifetime anxiety condition (*n* = 59) were compared with patients currently satisfying criteria for anxiety disorders (*n* = 26) and with patients previously affected by anxiety disorders, which failed to reach the diagnostic threshold at the time of the interview (*n* = 15). Gender, age, PD age of onset, illness duration, side of onset, PD type, presence of motor fluctuations, involuntary movements, gross neuroimaging alterations, and UPDRS (part III) score were not significantly different among patients receiving a previous (*n* = 15) or current diagnosis of anxiety disorders (*n* = 26) and patients without anxiety disorders (*n* = 59) (Supplementary Table S1). However, an almost significant relationship between current anxiety and hyperintensities or lacunes observed at neuroimaging emerged (Fisher’s exact *p* = 0.054). Indeed, while only one third of patients without anxiety disorders (16 of 59, 33.3%) and one third of patients with previous anxiety disorders (4 of 15, 33.3%) showed hyperintensities/lacunes, almost two thirds of patients with current anxiety disorders were affected by putative vascular lesions (14 of 26, 63.6%). Most differences among the groups were observed in neuropsychiatric and psychiatric features (Table 2).

Patients with current anxiety disorders showed significantly higher scores on the NMSS mood/apathy domain as compared with undiagnosed patients. Psychiatric symptoms before PD onset were significantly more frequent in patients with anxiety disorders than in those without anxiety. Similarly, lifetime major depression was significantly more frequent in both the groups with anxiety disorders, while was relatively infrequent in patients without current or lifetime anxiety disorders (8 of 59, 13.6%). However, significance was not reached when comparing current depressive symptoms severity measured by BDI-II among the groups (*p* = 0.061).

In contrast, robust significant differences at the PAS were observed, as expected. Both patients with previous and current anxiety disorders showed significantly higher scores on the PAS total score and persistent anxiety subscale (*p* < 0.001). Post-hoc contrasts showed no differences between current or previous anxiety disorders, while both the groups outscored the undiagnosed subgroup. Instead, there were no pairwise significant differences in episodic anxiety subscale between groups. Finally, the avoidant behavior subscale, as also the BPRS total score, significantly distinguished between patients without anxiety and patients with current anxiety disorders, while patients with previous anxiety disorders showed intermediate scores.

When looking at temperamental traits, no significant differences among the groups were observed in each TEMPS-M subscale, except than for the anxious temperament subscale. As expected, both patients with a history of anxiety disorders and patients with current anxiety disorders showed significantly higher scores than patients without anxiety disorders.

Both the anxiety groups also showed higher rates of lifetime antidepressant treatment and, specifically lifetime treatment with selective serotonin reuptake inhibitors (SSRIs), with marginally higher rates in patients with previous anxiety disorders. This latter group also showed higher rates of current psychiatric treatment and of lifetime treatment with tricyclic antidepressants (TCAs) compared with patients who never had any anxiety disorders. Patients with current anxiety, instead, showed intermediate rates of both the variables, and failed to significantly differ from both the other groups at the post-hoc comparisons.

### 3.3. Comparisons among Patients without Anxiety Disorders, with Lifetime Persistent Anxiety Disorders and with Lifetime Panic Disorders According to MINI-Plus

As previously mentioned, panic disorder was the most frequent lifetime anxiety comorbidity in our sample (*n* = 22). To evaluate differential correlates of a history of persistent and episodic anxiety, we compared patients without any lifetime anxiety disorders (*n* = 59), with lifetime persistent anxiety disorders (i.e., not panic disorder, *n* = 19) and with lifetime panic disorder (*n* = 22). Importantly, a great proportion of patients affected by panic disorder also showed other current or lifetime anxiety comorbidities (9 of 22, 40.9%). Eight subjects within the panic disorder group had lifetime agoraphobia, four of which currently satisfied diagnostic criteria for the disorder; two patients with panic and agoraphobia, also showed other anxiety comorbidity: one with social anxiety disorder, and the other with GAD and specific phobia. Finally, one other patient within the panic disorder group currently satisfied criteria for GAD. In contrast, only three patients in the persistent anxiety group (3 of 19, 15.8%) showed multiple anxiety comorbidities, two between GAD and specific phobias, the other between GAD and agoraphobia. However, rates of multiple anxiety comorbidity did not significantly differ among the groups (Fisher’s exact *p* = 0.098).

Gender, age, PD age of onset, illness duration, side of onset, PD type, presence of motor fluctuations, involuntary movements, gross neuroimaging alterations, and UPDRS (part III) score were not significantly different among patients with panic disorder, with other anxiety disorders or without any anxiety disorder (Supplementary Table S2). Even in this case, most of the differences among the groups were observed in neuropsychiatric and psychiatric features (Table 3).

The anxiety disorder subgroups did not differ significantly for any variables except than for the onset of psychiatric symptoms before PD onset, which was the rule in panic disorder patients (21 of 22, 95.5%) and was significantly less frequent in both the other groups, and for PAS episodic anxiety subscale. In fact, patients with panic disorder showed higher score at the PAS episodic subscale compared to those without anxiety and with persistent (but not panic) anxiety, instead both the anxiety subgroups had higher scores in PAS total score, persistent anxiety and avoidant behavior subscales compared to the subgroup without any lifetime anxiety disorder. Panic disorder patients also showed higher rates of lifetime unipolar depressive disorders in comparison with patients without any anxiety disorders, while the group with persistent anxiety had an intermediate rate.

When compared with patients without anxiety, those with panic disorders also showed significantly higher rates of general psychopathology as measured by BPRS. Conversely, significantly higher scores in the NMSS mood/apathy domain were observed in patients with persistent anxiety compared with non-comorbid patients. Similarly, though not significantly, the highest severity of depressive features, as measured by BDI-II, was reported in the persistent anxiety subgroup, with descending scores, in order, in the panic disorder subgroup and in the subgroup without anxiety disorders (*p* = 0.059).

Temperamental anxiety traits were equally represented in both the anxiety subgroups, which scored significantly higher at the TEMPS-M anxious temperament subscale than patients without anxiety disorders.

Finally, both the anxiety groups also showed higher rates of lifetime antidepressant treatment and, specifically lifetime treatment with SSRIs, with marginally higher rates in patients with lifetime panic disorder. This latter group also showed higher rates of current psychiatric treatment and of lifetime treatment with TCAs compared with patients who never had any anxiety disorders. Patients with persistent anxiety disorders, instead, showed an intermediate rate of current psychiatric treatment, and a low rate of lifetime treatment with TCAs.

### 3.4. Clinical Correlates of Anxiety Symptoms Measured by Parkinson Anxiety Scale (PAS)

Anxiety symptoms severity was found to be significantly associated with other non-motor symptoms domains and psychopathological constructs in correlational analyses (Table 4). Overall anxiety severity measured by PAS total score was significantly, but modestly correlated with NMSS total score, sleep/fatigue and attention/memory domains (r = 0.21–0.29). Stronger significant positive correlations were observed, instead, between PAS total score and NMSS mood/apathy domain, BPRS total score, BDI-II total score and TEMPS-M depressive and anxious temperament subscales (r = 0.42–0.59), while a weaker association with cyclothymic temperament was also identified (r = 0.27). As for PAS persistent anxiety, a similar pattern of significant correlations was observed. Indeed, strong associations with general psychopathology measured by BPRS and mood symptoms as assessed by NMSS mood/apathy domain and BDI-II were confirmed (r = 0.49–0.60). Depressive and anxious temperamental traits also showed significant positive correlations with persistent anxiety (r = 0.41–0.44), while the association with cyclothymic temperament was not significant. Episodic anxiety and avoidant behavior, instead, showed partially different correlates. PAS episodic anxiety subscale was significantly, but weakly, associated with NMSS total score, mood/apathy and gastrointestinal domains, BDI-II total score, and TEMPS-M depressive, cyclothymic and anxious temperament subscales (r = 0.21–0.25). Moderate correlations were found with BPRS total score (r = 0.43) and NMSS sleep/fatigue domain (r = 0.36). In addition, almost significant positive correlations with NMSS cardiovascular, perceptual problems and attention/memory domains were evidenced (r = 0.18–0.21, *p* < 0.10), while a negative association with cognitive performance at MMSE was suggested (r = −0.20, *p* = 0.059). Finally, PAS avoidant behavior only showed modest significant associations with BPRS total score, BDI-II total score, TEMPS-M depressive, cyclothymic and anxious temperament subscales (r = 0.20–0.28).

## 4. Discussion

In our study, around 41% of patients satisfied criteria for at least one lifetime diagnosis of anxiety disorder and more than 1 patient out of 4 was diagnosed with current anxiety disorders by means of MINI-Plus [23] according to DSM-TR-IV criteria [24]. These rates are consistent with both a previous report of 49% of PD patients satisfying criteria for at least one lifetime anxiety disorders [7] and a point prevalence of anxiety disorders of 31% reported in a recent meta-analysis [20]. The slightly lower rates of lifetime anxiety disorders we observed may be due to the fact that MINI-Plus does not investigate anxiety disorders not otherwise specified, which have been found to be highly prevalent in PD [13,20]. Overall, studies conducted to evaluate the prevalence of anxiety disorders in PD have shown a wide variability with rates ranging between 25% and 60% [35], probably as a result of the scarce homogeneity in settings, tools and sample selection (i.e., age, severity of PD, psychiatric evaluation).

In our sample, the most frequent lifetime anxiety disorder was panic disorder, with a prevalence reaching 22%, while only 3 patients had current panic disorder. Instead, 15% of the sample received the diagnosis of current GAD, that was the most frequent current anxiety disorder, as previously reported by the meta-analysis of Broen et al. [20]. The reduction in current panic disorder could be explained by the occurrence of atypical manifestations after the onset of PD, that may pass underdetected by traditional diagnostic approaches [7,36,37]. In this regard, panic-like manifestations related to “wearing off” phenomena and motor fluctuations often represent a diagnostic challenge [7].

In previous studies, anxiety disorders have been variably associated with rigid-akinetic phenotype [38], postural instability and gait disorder [8,39], nevertheless the evidence on relationships between anxiety and motor features is limited [40,41]. Some studies have linked the occurrence of anxiety in PD with younger age at onset [8,42], shorter duration of disease [43], greater disease severity [8], greater cognitive impairment [35,38], dysautonomic features [39] and motor fluctuations [42,44,45]. In our study, no significant associations between anxiety disorders and neurological variables were observed. However, an interesting trend toward the association between current anxiety disorders and hyperintensities/lacunes was found. Although the relationship among vascular risk, white matter hyperintensities and late-life depression vulnerability is well known [46], other neuropsychiatric syndromes of the geriatric age, including anxiety, can be favored and intensified by cerebrovascular ischemic damage [47,48,49]. Interestingly, an increased risk of anxiety has been observed in association with white matter abnormalities in Alzheimer’s disease patients [5] and our results support the hypothesis that this kind of alterations can affect circuits involved in anxiety regulation also in PD patients.

As expected, patients currently diagnosed with an anxiety disorder had a greater overall psychopathology compared to patients with previous anxiety and without anxiety disorders. Compared to the other groups, the group with current anxiety disorders had a significantly higher score in the NMSS mood/apathy domain, which assess depressed or flat mood, nervousness, lack of interest and motivation. This finding is consistent with previous studies showing high rates of co-occurrence of depression and anxiety in PD [50]. Indeed, the degree of comorbidity between anxiety and depression in PD patients outweighs that found in non-PD subjects [11,15] and the prevalence of anxiety disorders in depressed patients with PD is higher than that observed in depressed patients with other chronic debilitating illnesses [14,15]. Studies examining the occurrence of psychiatric disorders prior to PD, have found that PD patients have more frequent histories of depression and anxiety than controls [51,52]. These results suggest that depression and anxiety may be prodromes of PD, rather than reactive manifestions secondary to motor disability. Although it has not been established whether depression is the cause of anxiety or underlying neurochemical changes are responsible for both, it is possible that these disorders represent different facets of a unitary syndrome in PD. Importantly, in our sample, higher rates of comorbidity with lifetime unipolar depression were found both in patients with previous and current anxiety disorders compared to patients without anxiety, while current depressive symptoms severity was not significantly different among the groups. This could be due to multiple temporal relations between mood and anxiety symptoms. Indeed, PD-related anxiety disorders are prominent not only during major depression, but also prior to and after depressive episodes [14].

At the moment of the interview, patients with lifetime persistent anxiety showed more depressive symptoms compared to patients without anxiety. An association between depression and anxiety disorders was observed by some authors [15], however in other studies it was suggested that the high comorbidity rates previously found may be largely due to an artifact of the diagnostic system [53,54]. In particular, there is a strong overlap between the diagnostic criteria of GAD and those of depressive disorders. GAD symptoms such as insomnia, fatigue, impaired concentration and restlessness, in fact, also represent core diagnostic criteria of major depression. Even though the group with persistent anxiety had a higher load of depressive symptoms compared to the rest of the sample, the greatest psychiatric burden at the moment of interview seemed to belong to patients with lifetime panic disorder, which had a higher score at BPRS and were more frequently under psychiatric treatment compared to patients without anxiety. In most patients with panic disorder, differently from the other groups, psychiatric onset occurred prior to the neurological one. As a consequence, a longer history of psychiatric symptoms can be posited for patients with panic, which also explains the higher rate of lifetime comorbidity with depression compared to patients without anxiety.

Both patients with previous and current diagnoses of anxiety disorders showed higher levels of anxiety in PAS total score and persistent anxiety subscale compared to patients without anxiety disorders, providing an esteem of the power of PAS in positively identifying PD patients with significant anxiety symptoms at the moment of the interview, even when they had not satisfied criteria for a current diagnosis of anxiety disorders according to DSM-IV-TR. However, the group with current anxiety had the highest score in the PAS avoidant behavior subscale, which could reflect DSM-IV-TR requirements of avoidant behaviors to reach a diagnostic threshold. In other words, patients with previous anxiety disorders showed significant anxious distress but failed to be currently diagnosed due to the lack of avoidant behavior. Alternatively, we could posit that anxiety in PD patients mainly displays through avoidant behavior and situational anxiety, which could be easily triggered by ordinary experiences, given the disabilities associated with PD.

When patients were subdivided according to anxiety subtype (persistent vs. episodic), significantly higher scores in the PAS total, persistent and avoidant subscales were found in both the anxiety groups compared to patients without lifetime anxiety disorders. Although only 3 of 22 patients with lifetime panic disorders were currently diagnosed with panic at the moment of interview, the panic group had a higher score in the PAS episodic anxiety subscale compared to both group with persistent anxiety and without anxiety, suggesting that this subscale can efficiently detect PD patient with panic symptoms that are not properly recognized by the MINI-Plus. In other words, the PAS episodic anxiety subscale could fit well the manifestations of panic in the PD population, having the ability to identify those patients, previously affected by typical panic disorder, who currently show different atypical manifestations mediated by PD pathoplastic effects, which are less recognizable according to usual assessment instruments. As for PAS total score, TEMPS-M anxious temperament subscale was found to distinguish patients with anxiety disorders, whether persistent or episodic, current or not, from patients without anxiety, confirming the association of some personality traits and anxiety disorders in PD patients [42]. Importantly, psychiatric symptoms preceding PD onset were significantly more frequent in patients with anxiety compared to the group without anxiety disorder, allowing to hypothesize that in PD patients with anxiety, a constitutional vulnerability subtend psychiatric manifestations occurring during the whole lifetime.

Finally, patients with anxiety disorders showed higher rates of lifetime antidepressant and SSRIs treatment compared to patients who never had any anxiety disorders. However, among patients with anxiety disorders, the group with current anxiety had specifically lower frequency of lifetime treatment with TCAs compared to patients with previous anxiety. Indeed, despite their efficacy [55], the prescription of TCAs in PD patients is limited by anticholinergic side effects, that may be particularly troublesome for those subjects having an advanced disease or old age [56]. The lifetime use of TCAs was specifically greater in patients with lifetime panic disorder compared to patients without anxiety, probably as a consequence of the association between panic and lifetime depression. In fact, although TCAs also play a role in the treatment of anxiety disorders, are primarily used as antidepressants [57].

Several limitations of this study should be acknowledged. A first limitation comes from the clinical setting in which patients were recruited, a tertiary referral neurological unit, where comorbid patients could have been overrepresented, thus undermining the representativeness of the sample and, together with the limited sample size, the generalizability of the results to the whole PD population. Second, the cross-sectional study design restricted the assessment of lifetime psychiatric comorbidity to retrospective accounts, which may be at risk of recall bias, and limited the possibility to unravel temporal relationships between the occurrence of anxiety and other non-motor symptoms of PD. In addition, the evaluation of depressive symptoms severity and affective temperaments was based on self-report questionnaires, which may be biased by differences in social desirability, lack of insight or malingering attempts. Nevertheless, to avoid these risks, the assessment of motor and non-motor signs of PD, general cognition and psychopathology was based on clinician-rated instruments, namely UPDRS, NMSS and BPRS. Moreover, the observer-rated version of PAS was used to measure anxiety symptoms severity. Finally, no multiple comparison corrections of statistical analyses were applied. However, given the exploratory nature of our study and the limited subgroups size, this approach permitted to highlight potentially meaningful associations of interest. In any case, further studies are warranted in order to confirm or reject our findings.

Despite these limitations, the use of different assessment methods allowed to better characterize different dimensions of anxious symptomatology in PD. In addition, several analyses were performed, that permitted to unravel subtle differences among various anxious manifestations. Based on our results, we can confirm that a close relationship between anxiety disorders and PD exists, as previously highlighted [58]. Patients with anxious temperament features and a history of anxiety disorders, often comorbid with mood disorders and treated with antidepressants, are highly prevalent in PD samples and also more prone to develop significant anxiety and mood symptoms after PD onset. According to the characterization with PAS, anxiety in PD does not reflect a dichotomous scheme and manifestations of one dimension (i.e., persistent, episodic, avoidance) can be present into another, drawing up a peculiar anxiety profile. Persistent anxiety symptoms, in fact, are highly frequent even in PD patients with panic, as well as avoidant behaviors are shared by both patients with persistent and episodic anxiety. A higher specificity of PAS episodic anxiety subscale has been observed, whose highest score was reported in patients with lifetime panic disorder, independently from current DSM-based diagnosis. Overall, DSM-derived criteria for anxiety disorders could not perfectly fit the anxious phenomenology observed in PD patients, and the use of specifically designed instruments, such as the PAS, to assess anxiety subtypes and severity should be encouraged and warrant further investigation. The identification of anxious subjects among those with PD is fundamental, since non-motor symptoms have a high burden on the self-perceived health status of those patients [59,60], affecting greatly their daily function [9]. Even more than depression, anxiety contributes to worsening of well-being in patients [10,60,61] and caregivers’ stress [60]. Given the overall high impact of anxiety on patients’ quality of life [58,61,62], and the substantial improvement if early detected and treated [63], clinicians should not underestimate the extent of anxiety symptoms in PD.

## Figures and Tables

**Table 1:** Anxiety disorders in patients with Parkinson’s disease (*n* = 100).

Anxiety Disorders Comorbidity	*n* (%)
Anxiety disorders lifetime	41 (41%)
Anxiety disorders current	26 (26%)
Panic Disorder lifetime	22 (22%)
Panic Disorder current	3 (3%)
Agoraphobia lifetime	13 (13%)
Agoraphobia current	8 (8%)
Generalized anxiety disorder current	15 (15%)
Social anxiety disorder current	2 (2%)
Specific phobias current	4 (4%)

**Table 2:** Differences in non-motor symptoms and psychiatric features among PD patients without anxiety disorders, with previous or with current anxiety disorders.

	A. without Anxiety (*n* = 59)	B. Previous Anxiety (*n* = 15)	C. Current Anxiety (*n* = 26)			
Non-Motor Symptoms	M ± SD/*N* (%)	M ± SD/*N* (%)	M ± SD/*N* (%)	χ^2^	*p*	Post-Hoc
NMSS total score	32.59 ± 25.7	56.08 ± 48.64	34.88 ± 23.77	2.70	0.26	
NMSS cardiovascular	1.24 ± 2.61	1.38 ± 1.76	1.4 ± 3.65	1.50	0.47	
NMSS sleep/fatigue	4.73 ± 4.8	6.62 ± 7.77	7.04 ± 6.33	2.11	0.35	
NMSS mood/apathy	7.47 ± 11.71	11.38 ± 11.27	13.84 ± 13.82	7.33	**0.03**	A < C
NMSS perceptual problems	1.33 ± 2.7	2.54 ± 4.43	1 ± 2.4	1.16	0.56	
NMSS attention/memory	3.98 ± 6.98	5.54 ± 8.05	3.8 ± 5.14	0.49	0.78	
NMSS gastrointestinal	5.35 ± 6.36	8.54 ± 13.72	4.44 ± 4.74	0.03	0.99	
NMSS urinary	6.39 ± 6.62	12.46 ± 13.96	6.16 ± 6.37	2.14	0.34	
NMSS sexual function	4.25 ± 5.84	6.23 ± 8.29	3.68 ± 6.27	1.25	0.54	
NMSS miscellaneous	7.08 ± 7.54	12.77 ± 9.28	7.36 ± 9.08	5.46	0.07	
**Psychiatric features**						
Age of psychiatric onset	50.49 ± 19.45	39.2 ± 15.4	47.64 ± 18.52	4.11	0.13	
Symptoms before PD	23 (46%)	14 (93.3%)	19 (76%)	-	**0.00**	A < B, C
Unipolar depression *lifetime*	8 (13.6%)	7 (46.7%)	11 (42.3%)	11.67	**0.00**	A < B, C
Bipolar disorders *lifetime*	20 (33.9%)	4 (26.7%)	8 (30.8%)	-	0.91	
ICDs *lifetime*	19 (32.2%)	5 (33.3%)	10 (38.5%)	0.32	0.85	
**Psychiatric rating scales**						
PAS total score	6.38 ± 7.07	12.6 ± 7.84	16.65 ± 8.83	24.87	**0.00**	A < B, C
PAS persistent anxiety	4.68 ± 5.06	9 ± 5.52	10.81 ± 4.87	22.26	**0.00**	A < B, C
PAS episodic anxiety	0.57 ± 1.61	1.8 ± 2.14	2.58 ± 4.08	7.48	0.02	-
PAS avoidant behavior	1.12 ± 1.91	1.8 ± 2.81	3.27 ± 3.07	13.15	**0.00**	A < C
BPRS total score	32.55 ± 7.22	36.13 ± 8.18	36.81 ± 7.91	7.07	**0.03**	A < C
BDI-II total score	7.18 ± 6.72	10.2 ± 8.55	11.65 ± 8.68	5.59	0.06	
TEMPS-M depressive	11.61 ± 4.12	13.29 ± 5.06	14.92 ± 6.7	4.39	0.11	
TEMPS-M cyclothymic	10.91 ± 4.42	11.64 ± 5.69	11.48 ± 5.3	0.15	0.93	
TEMPS-M hyperthymic	19.79 ± 5.68	20.14 ± 5.78	17.4 ± 6.61	2.97	0.23	
TEMPS-M irritable	10.5 ± 4.88	11.57 ± 5.39	10.32 ± 4.02	0.27	0.87	
TEMPS-M anxious	10.79 ± 3.51	14.21 ± 4.06	13.68 ± 4.48	13.82	**0.00**	A < B, C
**Psychiatric treatment**						
Currently treated	29 (49.2%)	13 (86.7%)	19 (73.1%)	-	**0.01**	A < B
TCAs *lifetime*	6 (10.3%)	6 (40%)	6 (23.1%)	7.61	**0.02**	A < B
SSRIs *lifetime*	14 (24.1%)	11 (73.3%)	14 (53.8%)	-	**0.00**	A < B, C
TCAs or SSRIs *lifetime*	16 (27.1%)	12 (80%)	16 (61.5%)	-	**0.00**	A < B, C

Pearson’s χ^2^ and Kruskal-Wallis’ χ^2^ were respectively reported for categorical and continuous variables. Abbreviations: BDI-II = Beck Depression Inventory-II; BPRS = Brief Psychiatric Rating Scale; ICDs = Impulse Control Disorders; M = mean; NMSS = Non-Motor Symptoms Scale; PAS = Parkinson Anxiety Scale; PD = Parkinson Disease; SD = standard deviation; SSRIs = Selective Serotonin Reuptake Inhibitors; TCAs = Tricyclic Antidepressants; TEMPS-M = Temperament Evaluation of Memphis, Pisa, Paris and San Diego-Münster.

**Table 3:** Differences in non-motor symptoms and psychiatric features among PD patients without anxiety disorders, with lifetime persistent anxiety disorders or with lifetime panic disorders.

	A. without Anxiety (*n* = 59)	B. Persistent Anxiety (*n* = 19)	C. Panic Disorder (*n* = 22)			
Non-Motor Symptoms	M ± SD/*N* (%)	M ± SD/*N* (%)	M ± SD/*N* (%)	χ^2^	*p*	Post-Hoc
NMSS total score	32.59 ± 25.7	35.11 ± 25.42	48.45 ± 41.75	2.54	0.28	
NMSS cardiovascular	1.24 ± 2.61	0.56 ± 1.04	2.15 ± 4.07	3.68	0.16	
NMSS sleep/fatigue	4.73 ± 4.8	4.94 ± 5.34	8.65 ± 7.51	3.54	0.17	
NMSS mood/apathy	7.47 ± 11.71	13.89 ± 12.43	12.2 ± 13.57	7.64	**0.02**	A < B
NMSS perceptual problems	1.33 ± 2.7	0.67 ± 1.91	2.3 ± 4.01	1.46	0.48	
NMSS attention/memory	3.98 ± 6.98	2.78 ± 4.08	5.85 ± 7.49	2.13	0.35	
NMSS gastrointestinal	5.35 ± 6.36	4.78 ± 5.65	6.8 ± 11.15	0.01	1.00	
NMSS urinary	6.39 ± 6.62	6.28 ± 6.68	10.15 ± 12.03	1.16	0.56	
NMSS sexual function	4.25 ± 5.84	6.22 ± 7.61	3.05 ± 6.25	2.87	0.24	
NMSS miscellaneous	7.08 ± 7.54	8.89 ± 10.17	9.5 ± 8.88	1.11	0.57	
**Psychiatric features**						
Age of psychiatric onset	50.49 ± 19.45	47.67 ± 16.73	41.86 ± 18.42	2.98	0.23	
Symptoms before PD	23 (46%)	12 (66.7%)	21 (95.5%)	-	**0.00**	A, B < C
Unipolar depression *lifetime*	8 (13.6%)	7 (36.8%)	11 (50%)	12.49	**0.00**	A < C
Bipolar disorders *lifetime*	20 (33.9%)	6 (31.6%)	6 (27.3%)	0.33	0.85	
ICDs *lifetime*	19 (32.2%)	8 (42.1%)	7 (31.8%)	0.69	0.71	
**Psychiatric rating scales**						
PAS total score	6.38 ± 7.07	14.37 ± 6.55	15.86 ± 10.17	23.52	**0.00**	A < B, C
PAS persistent anxiety	4.68 ± 5.06	11.32 ± 4.77	9.14 ± 5.31	22.57	**0.00**	A < B, C
PAS episodic anxiety	0.57 ± 1.61	0.68 ± 1.95	3.68 ± 3.94	18.77	**0.00**	A, B < C
PAS avoidant behavior	1.12 ± 1.91	2.37 ± 2.41	3.05 ± 3.5	9.30	**0.01**	A < B, C
BPRS total score	32.55 ± 7.22	36 ± 7.92	37.05 ± 8.06	7.23	**0.03**	A < C
BDI-II total score	7.18 ± 6.72	12 ± 8.67	10.36 ± 8.59	5.67	0.06	
TEMPS-M depressive	11.61 ± 4.12	14.28 ± 5.44	14.38 ± 6.82	4.19	0.12	
TEMPS-M cyclothymic	10.91 ± 4.42	9.78 ± 3.42	13.05 ± 6.3	2.41	0.30	
TEMPS-M hyperthymic	19.79 ± 5.68	16.56 ± 5.64	19.95 ± 6.7	3.42	0.18	
TEMPS-M irritable	10.5 ± 4.88	9.17 ± 3.05	12.14 ± 5.17	2.64	0.27	
TEMPS-M anxious	10.79 ± 3.51	13.22 ± 4.26	14.43 ± 4.33	14.19	**0.00**	A < B, C
**Psychiatric treatment**						
Currently treated	29 (49.2%)	12 (63.2%)	20 (90.9%)	-	**0.00**	A < C
TCAs *lifetime*	6 (10.3%)	3 (15.8%)	9 (40.9%)	-	**0.01**	A < C
SSRIs *lifetime*	14 (24.1%)	10 (52.6%)	15 (68.2%)	14.69	**0.00**	A < B, C
TCAs or SSRIs *lifetime*	16 (27.1%)	11 (57.9%)	17 (77.3%)	18.20	**0.00**	A < B, C

Pearson’s χ^2^ and Kruskal-Wallis’ χ^2^ were respectively reported for categorical and continuous variables. Abbreviations: BDI-II = Beck Depression Inventory-II; BPRS = Brief Psychiatric Rating Scale; ICDs = Impulse Control Disorders; M = mean; NMSS = Non-Motor Symptoms Scale; PAS = Parkinson Anxiety Scale; PD = Parkinson Disease; SD = standard deviation; SSRIs = Selective Serotonin Reuptake Inhibitors; TCAs = Tricyclic Antidepressants; TEMPS-M = Temperament Evaluation of Memphis, Pisa, Paris and San Diego-Münster.

**Table 4:** Clinical Correlates of Anxiety Symptoms Measured by Parkinson Anxiety Scale (PAS).

	PAS Total Score		PAS Persistent Anxiety		PAS Episodic Anxiety		PAS Avoidant Behavior	
Neurological Rating Scales	r	*p*	r	*p*	r	*p*	r	*p*
UPDRS score (part III)	−0.04	0.72	−0.02	0.87	0.04	0.69	−0.05	0.66
MMSE total score	−0.11	0.30	−0.11	0.32	−0.20	0.06	−0.04	0.73
**Non-Motor Symptoms**								
NMSS total score	0.21	**0.04**	0.23	**0.03**	0.24	**0.03**	0.01	0.96
NMSS cardiovascular	0.17	0.11	0.11	0.29	0.18	0.09	0.08	0.47
NMSS sleep/fatigue	0.29	**0.01**	0.23	**0.03**	0.36	**0.00**	0.20	0.06
NMSS mood/apathy	0.50	**0.00**	0.59	**0.00**	0.23	**0.03**	0.17	0.11
NMSS perceptual problems	0.04	0.69	0.00	0.97	0.20	0.05	−0.01	0.94
NMSS attention/memory	0.21	**0.05**	0.25	**0.02**	0.21	0.05	0.04	0.71
NMSS gastrointestinal	0.16	0.13	0.19	0.08	0.21	**0.05**	−0.03	0.78
NMSS urinary	0.12	0.27	0.15	0.17	0.14	0.18	−0.07	0.54
NMSS sexual function	−0.05	0.66	0.01	0.89	0.02	0.82	−0.16	0.12
NMSS miscellaneous	0.05	0.61	0.08	0.43	0.03	0.77	−0.04	0.69
**Psychiatric Rating Scales**								
BPRS total score	0.59	**0.00**	0.60	**0.00**	0.43	**0.00**	0.28	**0.00**
BDI-II total score	0.44	**0.00**	0.49	**0.00**	0.25	**0.01**	0.20	**0.05**
TEMPS-M depressive	0.42	**0.00**	0.41	**0.00**	0.22	**0.03**	0.26	**0.01**
TEMPS-M cyclothymic	0.27	**0.01**	0.17	0.11	0.22	**0.03**	0.22	**0.03**
TEMPS-M hyperthymic	−0.03	0.76	−0.01	0.91	0.04	0.73	−0.08	0.45
TEMPS-M irritable	0.16	0.11	0.14	0.18	0.12	0.25	0.05	0.64
TEMPS-M anxious	0.47	**0.00**	0.44	**0.00**	0.25	**0.01**	0.26	**0.01**

Abbreviations: BDI-II = Beck Depression Inventory-II; BPRS = Brief Psychiatric Rating Scale; MMSE = Mini-Mental State Examination; NMSS = Non-Motor Symptoms Scale; PAS = Parkinson Anxiety Scale; TEMPS-M = Temperament Evaluation of Memphis, Pisa, Paris and San Diego-Münster; UPDRS = Unified Parkinson’s Disease Rating Scale.

## Data Availability

Data available on request due to restrictions eg privacy or ethical. The data presented in this study are available on request from the corresponding author.

## Supplementary Materials

The following are available online at https://www.mdpi.com/article/10.3390/jcm10112302/s1, Table S1: Differences in demographic and neurological features among PD patients without anxiety disorders, with previous or with current anxiety disorders, Table S2: Differences in demographic and neurological features among PD patients without anxiety disorders, with lifetime persistent anxiety disorders or with lifetime panic disorders.
