# Supplementary material for: Prevalence and Clinical Correlates of Comorbid Anxiety and Panic Disorders in Patients with Parkinson’s Disease
Source: J Clin Med. 2021 May 25;10(11):2302. doi: 10.3390/jcm10112302 (PMC8198165; doi:10.3390/jcm10112302)
Supplement: Supplementary file 1 [file jcm-10-02302-s001.zip › jcm-1188466-supplementary.pdf]

Table S1. Differences in demographic and neurological features among PD patients without anxiety disorders, with previous or with current anxiety disorders.

	**Without Anxiety (*n* = 59)**	**Previous Anxiety (*n* = 15)**	**Current Anxiety (*n* = 26)**		
**Demographic Variables**	**M ± SD/*N* (%)**	**M ± SD/*N* (%)**	**M ± SD/*N* (%)**	χ^2	*p*
Age	66.88 ± 11.07	64.33 ± 13.63	69.42 ± 6.71	1.39	0.50
Gender (females)	20 (33.9%)	9 (60%)	10 (38.5%)	3.43	0.18
**PD Features**					
Age of PD onset	58.61 ± 13.18	55.93 ± 12.6	62.54 ± 7.96	2.67	0.26
PD duration (years)	8.27 ± 5.62	8.4 ± 6.61	6.88 ± 6.72	2.17	0.34
Motor fluctuations	21 (36.2%)	5 (33.3%)	7 (26.9%)	0.70	0.71
Involuntary movements	21 (36.2%)	4 (26.7%)	8 (30.8%)	-	0.80
PD side of onset				-	0.24
Right	28 (66.7%)	5 (11.9%)	9 (21.4%)		
Left	21 (46.7%)	9 (20.5%)	14 (31.8%)		
Bilateral	1 (33.3%)	0 (0%)	2 (66.7%)		
PD type				-	0.36
Rigid-acinetic	25 (67.6%)	3 (8.1%)	9 (24.3%)		
Tremor	20 (47.6%)	9 (21.4%)	13 (31%)		
Mixed	10 (66.7%)	2 (13.3%)	3 (20%)		
**Gross neuroimaging Abnormalities**					
Hyperintensities/lacunes	16 (33.3%)	4 (33.3%)	14 (63.6%)	-	0.05
Enlargement of the ventricles/ hydrocephalus	2 (4.2%)	0 (0%)	3 (13.6%)	-	0.27
Atrophy	15 (31.2%)	3 (23.1%)	10 (45.5%)	-	0.38
**Neurological Rating Scales**					
UPDRS score (part III)	24.47 ± 9.74	22.36 ± 9.88	22.95 ± 9.84	0.83	0.66
MMSE total score	28.15 ± 3.12	28.79 ± 1.76	28.46 ± 1.91	0.79	0.67

Pearson's χ2 and Kruskal-Wallis' χ2 were respectively reported for categorical and continuous variables. Abbreviations: M = mean; MMSE = Mini-Mental State Examination; PD = parkinson disease; SD = standard deviation; UPDRS = Unified Parkinson's Disease Rating Scale.

Table 2. Differences in demographic and neurological features among PD patients without anxiety disorders, with lifetime persistent anxiety disorders or with lifetime panic disorders.

	**Without Anxiety (n = 59)**	**Persistent Anxiety (n = 19)**	**Panic Disorder (n = 22)**		
**Demographic Variables**	**M ± SD/*N* (%)**	**M ± SD/*N* (%)**	**M ± SD/*N* (%)**	χ^2	p
Age	66.88 ± 11.07	68.05 ± 6.2	67.14 ± 12.48	0.06	0.97
Gender (females)	20 (33.9%)	6 (31.6%)	13 (59.1%)	4.82	0.09
**PD Features**					
Age of PD onset	58.61 ± 13.18	60.68 ± 7.51	59.64 ± 12.34	0.23	0.89
PD duration (years)	8.27 ± 5.62	7.37 ± 7.82	7.5 ± 5.61	1.91	0.39
Dopamine-agonists *lifetime*	39 (66.1%)	15 (78.9%)	10 (45.5%)	-	0.08
Motor fluctuations	21 (36.2%)	6 (31.6%)	6 (27.3%)	0.61	0.74
Involuntary movements	21 (36.2%)	5 (26.3%)	7 (31.8%)	0.66	0.72
PD side of onset				-	0.11
Right	28 (66.7%)	7 (16.7%)	7 (16.7%)		
Left	21 (46.7%)	9 (20.5%)	14 (31.8%)		
Bilateral	1 (33.3%)	2 (66.7%)	0 (0%)		
PD type				-	0.20
Rigid-acinetic	25 (67.6%)	5 (13.5%)	7 (18.9%)		
Tremor	20 (47.6%)	9 (21.4%)	13 (31%)		
Mixed	10 (66.7%)	4 (26.7%)	1 (6.7%)		
**Gross neuroimaging Abnormalities**					
Hyperintensities/lacunes	16 (33.3%)	9 (60%)	9 (47.4%)	3.70	0.16
Enlargement of the ventricles/ hydrocephalus	2 (4.2%)	2 (13.3%)	1 (5%)	-	0.36
Atrophy	15 (31.2%)	5 (33.3%)	8 (40%)	0.48	0.78
**Neurological Rating Scales**					
UPDRS score (part III)	24.47 ± 9.74	23.24 ± 8.85	22.26 ± 10.66	1.10	0.58
MMSE total score	28.15 ± 3.12	28.76 ± 1.44	28.43 ± 2.13	0.27	0.87

Pearson's χ^2 and Kruskal-Wallis' χ^2 were respectively reported for categorical and continuous variables.
